# Antitumor Effect of Traditional Drugs for Neurological Disorders: Preliminary Studies in Neural Tumor Cell Lines

**DOI:** 10.1007/s12640-022-00606-3

**Published:** 2022-11-30

**Authors:** Kevin Doello, Cristina Mesas, Francisco Quiñonero, Ana R. Rama, Celia Vélez, Gloria Perazzoli, Raúl Ortiz

**Affiliations:** 1 Institute of Biopathology and Regenerative Medicine (IBIMER), Biomedical Research Center (CIBM), 18100 Granada, Spain; 2 Medical Oncology Service, Virgen de Las Nieves Hospital, 18014 Granada, Spain; 3 Instituto Biosanitario de Granada (Ibs.Granada), SAS-Universidad de Granada, 18012 Granada, Spain; 4 Department of Anatomy and Embryology, University of Granada, 18071 Granada, Spain; 5 Department of Health Sciences, University Jaén, 23071 Jaén, Spain; 6 Department of Medicine, Physiotherapy and Nursing, University of Almería, 04120 Almería, Spain

**Keywords:** Glioblastoma, Levomepromazine, Haloperidol, Lacosamide, Valproic acid, Levetiracetam, Glatiramer acetate, Fingolimod, Biperiden, Dextromethorphan

## Abstract

Glioblastoma multiforme is the most common malignant primary brain tumor in adults. Despite new treatments developed including immunomodulation using vaccines and cell therapies, mortality remains high due to the resistance mechanisms presented by these tumor cells and the function of the blood–brain barrier that prevents the entry of most drugs. In this context of searching for new glioblastoma therapies, the study of the existing drugs to treat neurological disorder is gaining great relevance. The aim of this study was to determine, through a preliminary in vitro study on human glioblastoma (A172, LN229), anaplastic glioma (SF268) and neuroblastoma (SK-N-SH) cell lines, the possible antitumor activity of the active principles of several drugs (levomepromazine, haloperidol, lacosamide, valproic acid, levetiracetam, glatiramer acetate, fingolimod, biperiden and dextromethorphan) with the ability to cross the blood–brain barrier and that are commonly used in neurological disorders. Results showed that levetiracetam, valproic acid, and haloperidol were able to induce a relevant synergistic antitumor effect when associated with the chemotherapy currently used in clinic (temozolomide). Regarding the mechanism of action, haloperidol, valproic acid and levomepromazine caused cell death by apoptosis, while biperiden and dextromethorphan induced autophagy. Fingolimod appeared to have anoikis-related cell death. Thus, the assayed drugs which are able to cross the blood–brain barrier could represent a possibility to improve the treatment of neural tumors, though future in vivo studies and clinical trials will be necessary to validate it.

## Introduction

Among neural tumors, glioblastoma or glioblastoma multiforme is the most common malignant primary brain tumor in adults (45.2%) (Kanderi and Gupta 2022) with a worldwide incidence between 0.59 and 5 cases per 100,000 and a progressive increase due to the aging population among other factors. The median age at diagnosis is 62 years and the average survival is approximately 14.6 months (Grech et al. 2020) being one of the most aggressive tumors. Usually, their location is supratentorial (frontal, temporal, parietal and occipital lobes) and it is rarely located in the cerebellum. Currently, tumor surgical resection with maximum safety followed by radiotherapy and concurrent chemotherapy with Temozolamide is the main treatment (Kanderi and Gupta 2022). Other novel treatments include boron neutron capture therapy (BNCT), anti-angiogenic therapy, gene and epigenetic therapy, and the use of oncolytic viruses (Zhang et al. 2019). In addition, new therapeutic strategies including immunomodulation for glioblastoma using vaccines, cell-based therapies, and therapies that regulate immune checkpoints are being developed (Reardon et al. 2020; Wen et al. 2020; Medikonda et al. 2021).

In this context of searching for new glioblastoma therapies, strategies based on the existing drugs to treat neurological disorder have been analyzed. In fact, antipsychotics have shown promising results, mainly because they are able to cross the blood–brain barrier (Kamarudin and Parhar 2019). In addition, Lee et al. (2016) expose that anticonvulsivants such as oxacarbazepine or valproic acid show antitumor activity against glioblastoma cell lines and enhances temozolomide cytotoxicity (Lee et al. 2016). Moreover, Yamada et al. (2020) demonstrated the antitumor effect of riluzole, a glutamate receptor inhibitor, against glioblastoma cell lines by MGMT suppression and temozolomide sensibilization (Yamada et al. 2020).

Finally, in the last 30 years, the survival rates of glioblastoma patients have shown no improvement. The future of its treatment will depend on clinical and genomic studies that allow us to understand the origin and development of this complex disease. Meanwhile, the search for new treatments that improve the prognosis of these patients is necessary. The aim of this manuscript was to determine, through a preliminary in vitro study, the possible antitumor activity of the active principles of several drugs with the ability to cross the blood–brain barrier and that are commonly used in neurological disorders.

## Materials and Methods

### Reagents

Levomepromazine, haloperidol (antipsychotic), lacosamide, valproic acid, levetiracetam (antiepileptic), glatiramer acetate, fingolimod (immunomodulators), biperiden (antiparkinsonian) and dextromethorphan (cough treatment) were obtained from Sigma-Aldrich (Sigma, Saint Louis, Missouri, USA).

### Cells and Cell Culture

Human glioblastoma (A172, LN229), anaplastic glioma (SF268) and neuroblastoma (SK-N-SH) cell lines were obtained from American Type Culture (ATCC) and Scientific Instrumentation Center (CIC, Granada University, Granada, Spain). Both SF268 and SK-N-SH cells showed resistance to temozolomide (TMZ) through O-6-Methylguanine-DNA Methyltransferase (MGMT) overexpression. All cells lines were grown in Dulbecco’s Modified Eagle’s Medium (DMEM) (Sigma-Aldrich, Madrid, Spain) supplemented with 10% heat-inactivated fetal bovine serum (FBS) (Gibco, Madrid, Spain) and antibiotics (gentamicin/amphotericin-B + penicillin/streptomycin) (Sigma Aldrich, Madrid, Spain) at 1% and maintained in an incubator at 37 °C and 5% CO_2_ humidified atmosphere.

### Cell Treatment

Cells were treated with different compounds in monotherapy for 72 h at increasing concentrations, levomepromazine (1–200 µM), haloperidol (1–200 µM), biperiden (1–200 µM), fingolimod (1–200 µM), dextromethorphan (1–200 µM), lacosamide (1–50,000 µM), valproic (1–10,000 µM), levetiracetam (1–50,000 µM), glatiramer acetate (1–10,000 µM), TMZ (1–750 µM). All these compounds were obtained from Sigma Aldrich (Madrid, Spain). In the case of combination between TMZ (IC25) and other compounds (IC30), both were added at the same time to cell culture for 72 h.

### Cell Viability Assay

To investigate the effect of active principles on human glioblastoma cell proliferation, SF-268, A-172, SK-NSH (5 × 10^3^ cells/well) and LN-226 (8 × 10^3^ cells/well) were seeded in 48-well plates and incubated overnight. After 24 h, cell cultures were exposed to increasing concentrations of active principles (72 h) determining cell viability. Cells were fixed with 10% trichlooacetic acid (TCA) (20 min at 4 °C). Once dried, the plates were stained with 0.4% sulforhodamine B (SRB) in 1% acetic acid (20 min, in agitation). After three washes with 1% acetic acid, SRB was solubilized with Trizma ^®^ (10 nM, pH 10.5). Finally, the optical density (OD) was measured at 492 nm in a spectrophotometer EX-Thermo Multiskan. Cell survival (%) was calculated according to the following equation: Cell survival (%) = Treated cells OD – blank/Control OD − blank × 100. In addition, half maximal Inhibitory Concentration (IC50) was calculated (GraphPad Prism 6 Software, La Jolla, CA, USA). For the combination effect, the combination index (CI) was calculated using the Compusyn software (Chou and Martin 2005), where a CI > 1 indicates antagonism, where a CI level of < 1 indicates synergy and a CI level equal to 1 indicates additivity. IC30 doses of the studied compounds and IC25 doses of temozolomide were used for combination studies.

### Wound-Healing Assay

To determine the tumor cell migration capacity of cell lines and, therefore, their invasiveness and ability to generate metastases, an in vitro migration assay was performed with SF-268 cell line. Cells were seeded in 12-well plates with 3 × 10^5^ cell/well in 1 mL of culture medium and grown to 100% confluence in standard culture conditions. Once confluence was reached, a “wound” was made with a 100 µL sterile pipette tip followed by washing with PBS to remove the detached cells and the medium was substituted for serum-free DMEM. Immediately, cells were exposed to the active principles (non-cytotoxic dose, IC5-IC15) for 72 h. Images were taken with an inverted light microscope Olympus CKX41 (Olympus Corporation) at different times (0, 8, 24, 48, and 72 h) to observe cell migration in comparison to the control (cells without treatment). To evaluate the effect of the active principles, the percentage of migration was calculated by measuring the area free of tumor cells at different times (Image J software).

### Tunel Assay

To detect the apoptosis based on the detection of single- and double-stranded DNA breaks a tunel assay was carried out. SF-268 (3 × 10^4^ cells) were seeded in 8 well culture slides (Corning, USA), exposed to active principles (IC_50_) for 48 h, fixed with 100 µL of paraformaldehyde (4% in PBS, pH 7.4) and incubated 60 min at 25 °C. Then, cells were washed with PBS and resuspended with 100 µL/well permeabilisation solution (0.1% Triton X-100 in 0.1% sodium citrate) for 2 min on ice at 4 °C. Cells were washed again with PBS, resuspended in 50 µL/wll Tunel reaction mixture (Sigma Aldrich, Madrid, Spain) and incubated 1 h at 37 °C in a humidified atmosphere in the dark. Finally, cells were washed with PBS and observed by fluorescence microscopy (Leica Microsystems, Wetzlar, Germany).

### Lysotracker Labeling

To determine the apoptosis by autophagy, SF-268 (3 × 10^4^ cells) were seeded in 8 well culture slides (Corning, USA), exposed to active principles (IC_50_) for 24 h and stained and loaded with LysoTracker^®^ Red DND-99 (Thermo Fisher Scientific, Waltham, MA, USA) 50 nM for 30 min at 37 °C, a fluorescent red dye used for labeling and monitoring of acidic organelles. Cells were washed again with PBS and stained with Hoechst (1:1000). Finally, cells were observed under fluorescence microscopy (Leica Microsystems, Wetzlar, Germany).

### Statistical Analysis

Statistical analysis was performed by IBM SPSS Statistics 26.0 and GraphPad Prism 8. All the data were presented as the mean value with standard deviation (SD). All experiments were performed in triplicate. After the homogeneity test of variance, *t*-test was performed to compare the differences between groups with equal variance, while *F*-test was used for groups with uneven variance. Significance values were denoted by (*) *p* < 0.05 significant; (**) *p* ≤ 0.01 highly significant.

## Results

### Antiproliferative Activity in Cultured Cells

As shown in Table 1, the active principles analyzed showed very different antitumor effects on human glioblastoma lines although in all cases this effect was dose-dependent. Thus, levomepromazine was the drug that showed the lowest IC50 values in the four cell lines SF-268 (16 µM), SK-N-SH (23.3 µM), A-172 (22.7 µM) and LN-229 (50 µM) followed by the agent fingolimod that presented IC50 values of 16.6, 29.3, 20.3 µM and 23.6 µM in SF-268, SK-N-SH, A-172 and LN-229, respectively. Some agents showed no or very low effect such as lacosamide, valproic, levetiracetam and glatiramer acetate while others had a moderate or low effect such as haloperidol, biperiden and dextromethorphan.

**Table 1:** IC50 values of different drugs on glioblastoma cell lines

	**Ic50 (µM)**			
	**SF-268**	**SK-N-SH**	**A-172**	**LN-229**
Levomepromazine	16 ± 1.9	23.3 ± 2.1	22.77 ± 0.8	50 ± 1.1
Lacosamide	-	-	-	-
Valproic	9342.43 ± 3.2	7586.96 ± 2.8	6161.41 ± 1.9	9793.63 ± 2.2
Levetiracetam	25,670.65 ± 1,8	42,285.71 ± 1.5	34,795.32 ± 2.5	44,338.03 ± 1.7
Glatiramer acetate	1927.65 ± 0.6	2757.94 ± 0.8	2666.67 ± 1.3	2188.76 ± 2.2
Haloperidol	71.83 ± 0.3	67.14 ± 1.6	70.28 ± 0.9	38.47 ± 2.1
Biperiden	85.07 ± 1.4	86.84 ± 2.4	85.63 ± 0.3	97.28 ± 1.1
Fingolimod	16.55 ± 0.5	29.26 ± 2.5	20.26 ± 2.4	23.61 ± 3.1
Dextromethorphan	48.46 ± 4.2	157 ± 2.3	171.99 ± 3.9	86.5 ± 1.9

### Active Principles Enhanced the Temozolomide Antiproliferative Effect in SF-268 Cells

**Fig. 1:**
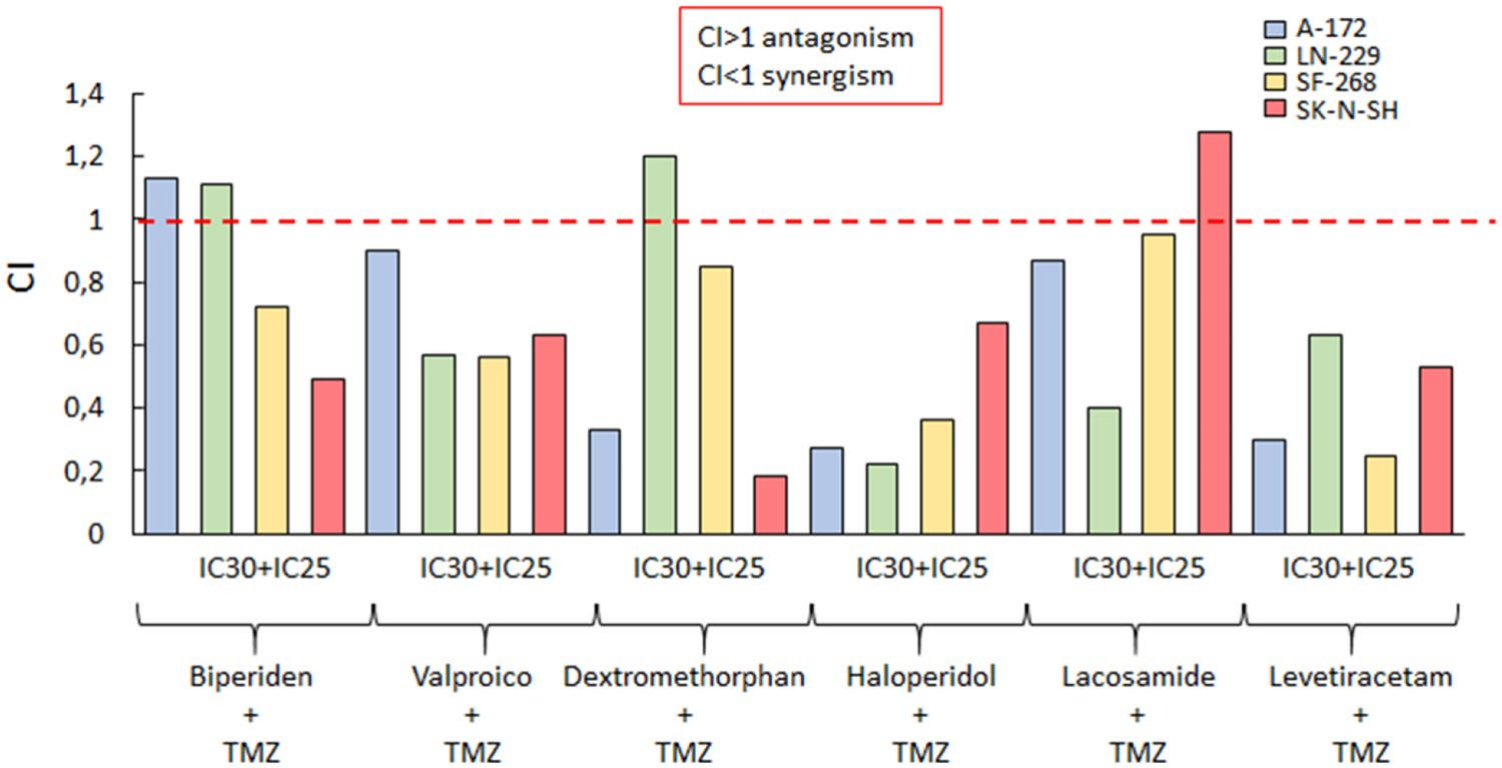
Combination indexes of different compounds with TMZ in different neural tumors cell lines. Combination index (CI) was calculated using the Compusyn software, where a CI > 1 indicates antagonism, where a CI level of < 1 indicates synergy and a CI level equal to 1 indicates additivity. IC30 doses of the studied compounds and IC25 doses of temozolomide were used for combination studies

As shown in Fig. 1, the active principles of some drugs combined with TMZ induced synergistic effects compared to monotherapies (CI < 1). The greatest synergistic effect was dependent not only on the used drug but also on the cell line analyzed. In fact, dextromethorphan, haloperidol, and levetiracetam in combination with TMZ showed the highest antiproliferative activity and synergistic effect on the A-172 cell line. Similarly, haloperidol and lacosamide + TMZ showed the greatest effect on the LN-229 cell line. Regarding the SF-268 cell line, haloperidol and levetiracetam associated with TMZ showed the highest synergistic activity. Finally, in the SK-N-SH cell line, dextromethorphan + TMZ showed the highest antiproliferative and synergistic effect. Valproic acid, haloperidol and leveraracetam showed a synergistic effect with TMZ in all studied cell lines.

### Analysis of Cell Migration

**Fig. 2:**
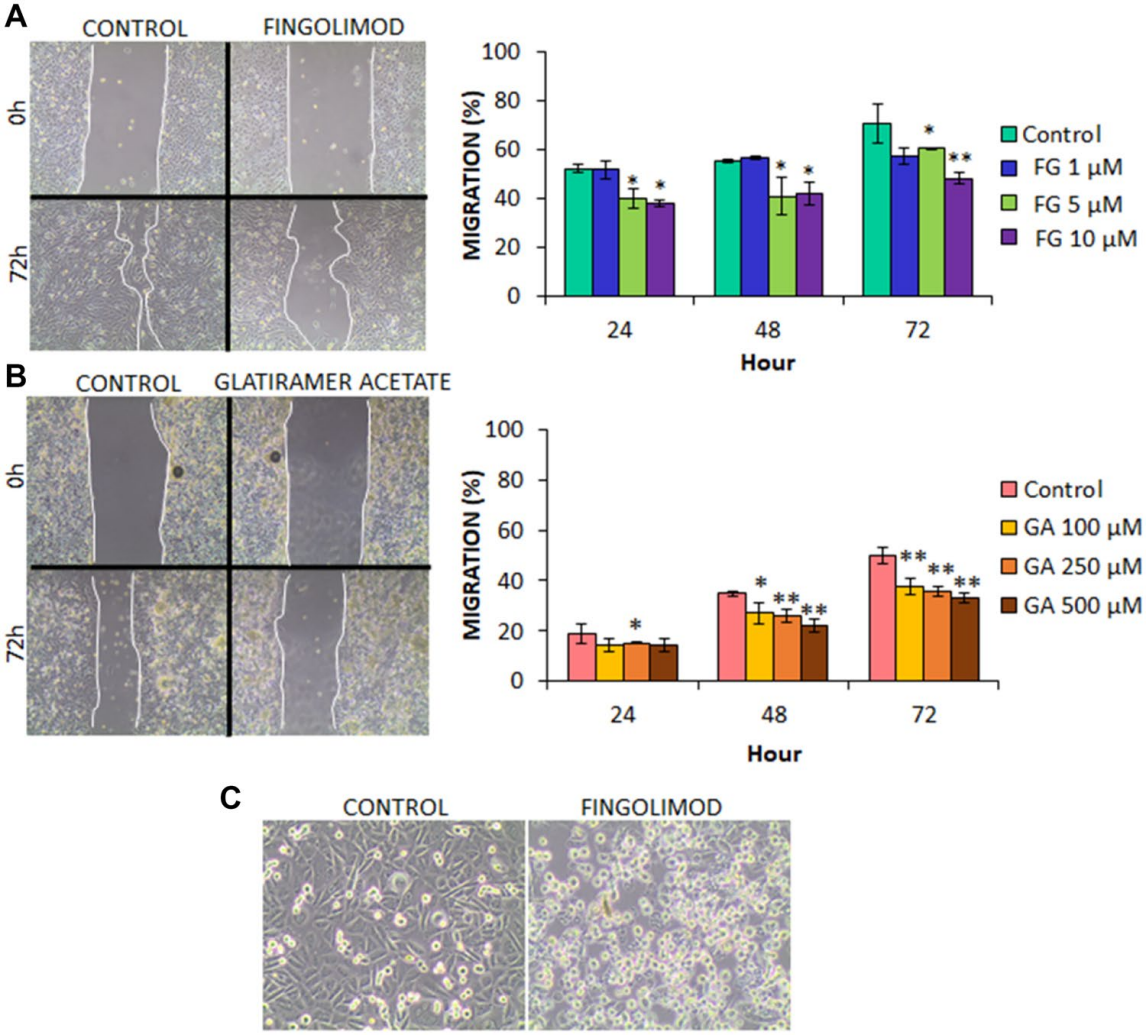
Wound healing assays results with **A** fingolimod and **B** glatiramer acetate in SF268 cell line. **C** Cell death induced by fingolimod with morphological changes compatible with anoikis in SF268 cell line

To assess the effects of the glatiramer acetate and fingolimod on tumor cell migration capacity, the migration of SF-268 cultured cells were analyzed by a cell wound healing assay. Results showed that non-cytotoxic doses of both drugs induced a decrease in tumor cell migration vs. control (non-treated) cells. In fact, the modulation of migration could be detected as early at 24 h after wound induction. However, the most significant reduction of cell migration was detected at 72 h (*p* < 0.05) (Fig. 2).

### Molecular Analysis of Cells Death Induction by Active Principles

**Fig. 3:**
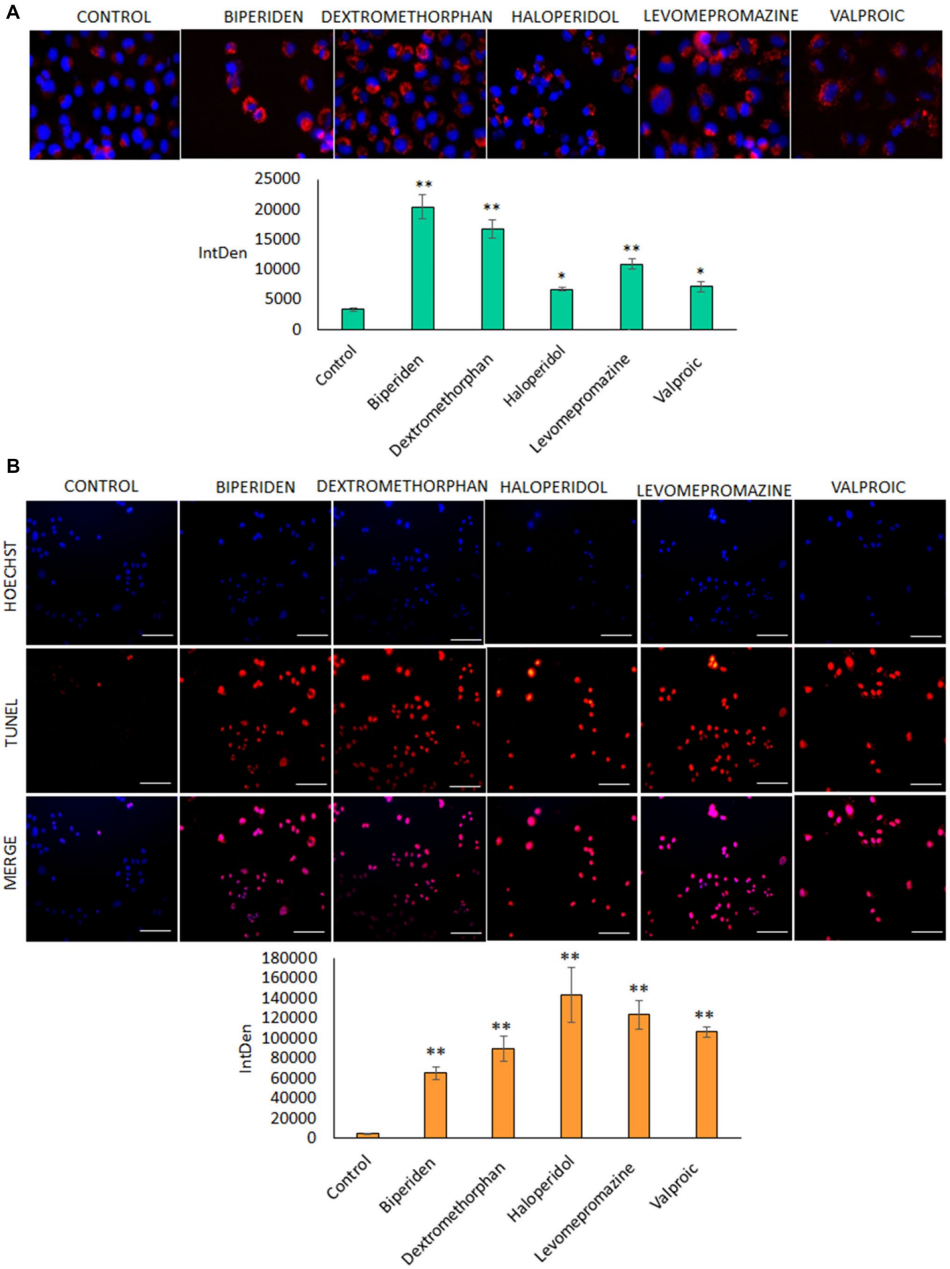
Mechanism of cells death by active principles. **A** TUNEL assay in SF268 cell line with different drugs. Fluorescence images and intensity graphics. (*) *p* < 0.05 significant; (**) *p* ≤ 0.01 highly significant, (***), *p* ≤ 0.001 very highly significant. **B** Lysotracker assays in SF268 cell line with different drugs. Fluorescence images and intensity graphics. (*) *p* < 0.05 significant; (**) *p* ≤ 0.01 highly significant, (***), *p* ≤ 0.001 very highly significant

Tunel assay was conducted to determine apoptosis mechanisms of the active principles. As shown in Fig. 3, haloperidol, levomepromazine and valproic were the drugs that induced a larger population of in SF-268 cells entering apoptosis (*p* < 0.05). On the other hand, Lysotracker assays determined that biperiden and dextromethorphan were the only drugs that induce a significant formation of autophagic vesicles in these same cells (*p* < 0.001).

## Discussion

Despite improvements in neurosurgical techniques, radiotherapy, development of new chemotherapeutics agents and the comprehensive genomic and molecular characterization, GBM remains one of the most aggressive brain tumors. In recent years, the possible antitumor effect exerted by some drugs used for neurological disorders that could help the treatment of GBM is gaining interest.

Our results showed that levomepromazine and fingolimod were the drugs with a higher antitumor effect reaching an IC50 between 16 and 50 µM in the first case and 16 µM and 23 in the second case, in neural tumor cell lines tested. In the case of levomepromazine, the antitumor activity could be explained by its binding and inhibition of translationally controlled tumor protein (TCTP), which is a novel target for differentiation therapy because it is down-regulated in tumor reversion experiments (Seo and Efferth 2016; Tripathi et al. 2021). On the other hand, the fingolimod effect may be related to the inhibition of multifunctional sphingosine-1-phosphate (S1P), a molecule involved oncogenesis, proliferation, migration and metastasis and that is overexpressed in GBM. In fact, this target has been described as a new therapeutic strategy in GBM (Mahajan-Thakur et al. 2017). In addition, the fingolimod cell death mechanism could be related to anoikis as suggested in our results showing detached and rounded cells after treatment.

Interestingly, some active principles not only exerted an antitumor effect against neural tumor cells but also showed a synergistic effect when associated with TMZ. Such is the case of haloperidol, levetiracetam and valproic in all studied cell lines, and, dextromethorphan in the A-172 and SK-N-SH cell lines or biperiden in SF268 and SK-N-SH cell lines. In our results, biperiden and dextromethorphan mainly induced autophagic cell death and valproic, haloperidol and levomepromazine, apoptotic cell death. It is known that haloperidol was able to induce apoptosis and increase caspase-8 expression in other glioblastoma cell lines (Papadopoulos et al. 2020). On the other hand, dextromethorphan could exert its antitumor effect through its activity as an agonist of ơ1 receptors that are overexpressed in many tumors of neural and non-neural origin (Mégalizzi et al. 2007). In addition, previous studies showed that levetiracetam inhibited tumor cell growth by decreasing the expression of O6-methylguanine-DNA methyltransferase (MGMT). This MGMT modulation may increase the p53 protein binding to the promoter region of the gene coding for that enzyme, through recruitment of the mSin3A/histone deacetylase 1 (HDAC1) complex. Thus, our results suggest that levetiracetam could increase the GBM sensitivity to temozolomide, with a consequent potential therapeutic benefit (Roh et al. 2020). In fact, Pallud et al. (2022) published an observational study in which the intake of oral levetiracetam during glioblastoma chemoradiation with temozolomide increased significantly overall survival in those patients (Pallud et al. 2022).

Finally, our study also highlights the ability of glatiramer acetate and fingolimod to inhibit GBM cell migration. This effect is probably related to their activity on cell adhesion receptors to the extracellular matrix (Mathias et al. 2017). Glatiramer acetate is a myelin receptor inhibitor and fingolimod is a sphingosine receptor blocker. In addition, biperiden produced, mainly, autophagy in glioblastoma cells, which could be explained by blocking MALT1 protease activity, which increases autophagy and culminates in lysosome-mediated cell death and concomitantly with the inactivation of mTOR (Jacobs et al. 2020).

## Conclusion

In conclusion, our results demonstrated that some traditional neurological compounds, such as neuropleptics, antiepileptics or immunomodulators, among others, showed significant antitumor activity against neural tumor cell lines in vitro. Furthermore, some of them (especially levetiracetam, valproic acid, and haloperidol) were able to induce a relevant synergistic antitumor effect when associated with TMZ. Regarding the mechanism of action, haloperidol, valproic acid and levomepromazine caused cell death by apoptosis, while biperiden and dextromethorphan induced autophagy. Fingolimod appears to have anoikis-related cell death. Thus, the assayed drugs which are able cross the blood–brain barrier, represents a possibility to improve the treatment of neural tumors. Future in vivo studies and clinical trials will be necessary to validate this new use.

## Data Availability

The datasets used can be accessed on request from the corresponding author.
